# TRUE Gene Silencing

**DOI:** 10.3390/ijms23105387

**Published:** 2022-05-11

**Authors:** Masayuki Nashimoto

**Affiliations:** Research Institute for Healthy Living, Niigata University of Pharmacy and Applied Life Sciences, Niigata 956-8603, Japan; mnashimoto@nupals.ac.jp; Tel.: +81-250-25-5118

**Keywords:** TRUE gene silencing, sgRNA, tRNase Z^L^, RNA therapeutics, multiple myeloma, tumor-associated macrophage

## Abstract

TRUE gene silencing is an RNA-mediated gene expression control technology and is termed after tRNase Z^L^-utilizing efficacious gene silencing. In this review, I overview the potentiality of small guide RNA (sgRNA) for TRUE gene silencing as novel therapeutics. First, I describe the physiology of tRNase Z^L^ and cellular small RNA, and then sgRNA and TRUE gene silencing. An endoribonuclease, tRNase Z^L^, which can efficiently remove a 3′ trailer from pre-tRNA, is thought to play the role in tRNA maturation in the nucleus and mitochondria. There exist various small RNAs including miRNA and fragments from tRNA and rRNA, which can function as sgRNA, in living cells, and human cells appear to be harnessing cytosolic tRNase Z^L^ for gene regulation together with these small RNAs. By utilizing the property of tRNase Z^L^ to recognize and cleave micro-pre-tRNA, a pre-tRNA-like or micro-pre-tRNA-like complex, as well as pre-tRNA, tRNase Z^L^ can be made to cleave any target RNA at any desired site under the direction of an artificial sgRNA that binds a target RNA and forms the pre-tRNA-like or micro-pre-tRNA-like complex. This general RNA cleavage method underlies TRUE gene silencing. Various examples of the application of TRUE gene silencing are reviewed including the application to several human cancer cells in order to induce apoptosis. Lastly, I discuss the potentiality of sgRNA as novel therapeutics for multiple myeloma.

## 1. Introduction

After a long-term struggle for their development, six antisense oligonucleotide therapeutics and two siRNA therapeutics for several diseases have been approved by US Food and Drug Administration [1,2,3], and this encourages us to further develop novel oligonucleotide therapeutics for various diseases. In this review, I overview the potentiality of small guide RNA (sgRNA) for RNA-mediated gene expression control technology, TRUE gene silencing (after tRNase Z^L^-utilizing efficacious gene silencing), as novel therapeutics. sgRNA guides target mRNA cleavage by tRNase Z^L^ as antisense oligonucleotide and siRNA guide target mRNA cleavage by RNase H1 and Argonaute 2, respectively.

Since TRUE gene silencing has been studied by only us and a few collaborators of us, this review focuses primarily on our studies. First, I describe the physiology of tRNase Z^L^ and cellular small RNA in Section 2, and then sgRNA and TRUE gene silencing in Section 3. In Section 3, I review four types of sgRNA, how to choose target sites for sgRNA, and examples of application of TRUE gene silencing, and discuss the potentiality of sgRNA as novel therapeutics for multiple myeloma in Section 3.3.

Although the development of sgRNA is still in its infancy, I expect that this review will stimulate readers’ interest in TRUE gene silencing to bring breakthroughs in sgRNA therapeutics for various diseases. Lastly, I would like to note that “sgRNA” in this review, which has been used since 2000, is completely different from sgRNA for the CRISPR/Cas9 system, to avoid confusion.

## 2. Gene Regulation by tRNase Z^L^ and Cellular Small RNA

### 2.1. Human tRNase Z

Human tRNase Z (tRNA 3′ processing endoribonuclease, 3′ tRNase, or RNase Z) (EC 3.1.26.11) is encoded on chromosomes 17 and 18. The long form (tRNase Z^L^) that consists of 826 amino acids is on the former and the short form (tRNase Z^S^) that consists of 363 amino acids is on the latter [4]. tRNase Z^L^, which can efficiently remove a 3′ trailer from pre-tRNA, is thought to play a role in tRNA maturation in the nucleus and mitochondria [4], while tRNase Z^S^, which can efficiently remove a phosphate from the CCA-less tRNA ending with the 2′,3′-cyclic phosphate, is thought to play a role in tRNA recycling in cytosol [5].

### 2.2. Targeted RNA Cleavage by tRNase Z^L^ under the Direction of sgRNA

**Figure 1:**
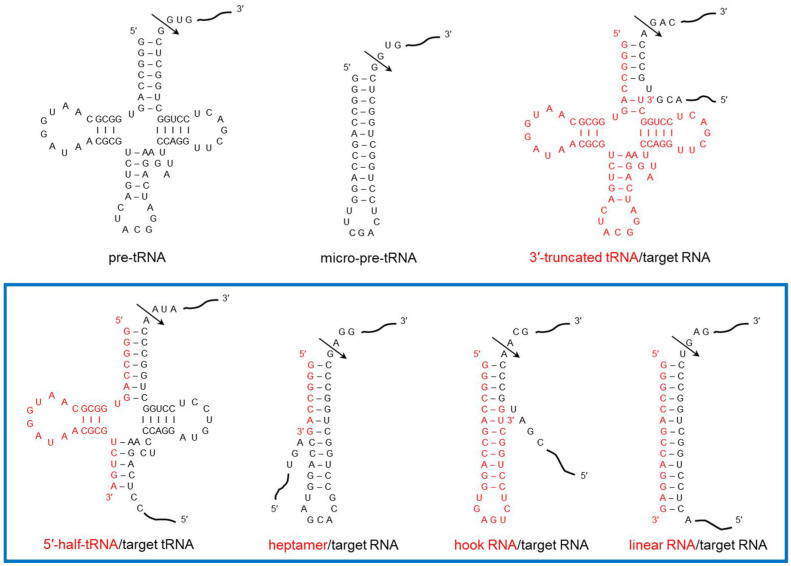
Substrates of tRNase Z^L^. The RNA complexes of the four types of sgRNA and their target RNAs are shown in a blue box. Arrows denote cleavage sites by tRNase Z^L^.

tRNase Z^L^ can recognize and cleave micro-pre-tRNA, a pre-tRNA-like or micro-pre-tRNA-like complex, as well as pre-tRNA [6]. By utilizing this property, tRNase Z^L^ can be made to cleave any target RNA at any desired site under the direction of an artificial sgRNA that binds a target RNA and forms the pre-tRNA-like or micro-pre-tRNA-like complex (Figure 1). There exist various small RNAs including miRNA and fragments from tRNA and rRNA, which can function as sgRNA, in living cells, and human cells appear to be harnessing cytosolic tRNase Z^L^ for gene regulation together with these small RNAs [7,8].

It has been shown that a 5′-half-tRNA^Glu^ and a 28S rRNA fragment in HEK293 cells, which co-immunoprecipitate with tRNase Z^L^, can function as sgRNA for tRNase Z^L^ and that these RNA fragments downregulate the PPM1F and DYNC1H1 mRNAs, respectively, in HEK293 cells [7]. Furthermore, the transcriptome analysis for HEK293 cells that overexpress tRNase Z^L^ has suggested that tRNase Z^L^ is likely to be involved in the p53 signaling pathway and apoptosis.

Various 3′-truncated tRNAs of ~67 nt have been found to exist as relatively stable complexes with tRNase Z^L^ in mouse FM3A cell extracts [9,10,11,12,13]. These RNA/enzyme complexes (generally termed RNase 65) have been shown to function as four-base-recognizing RNA cutters, in which the 4-nt 5′-terminal sequences of the 3′-truncated tRNAs are used to recognize target RNAs via 4 base-pairings (Figure 1). Although little is known about the physiological role and substrate of RNase 65, it has been shown that the level of a 3′-truncated tRNA^Arg^ increases in human BJAB cells after adding 5-fluorouracil [7].

It has been reported that various 5′-half-tRNAs and 3′-truncated tRNA^Gly^ and tRNA^Val^ exist in mouse epididymis and that the 5′-half-tRNA^Gly^ suppresses genes associated with the endogenous retroelement MERVL [14]. Although these 5′-half-tRNAs and 3′-truncated tRNAs should work as sgRNAs for tRNase Z^L^, it remains to be seen what their cellular target RNAs are and whether they can guide the target RNA cleavages by tRNase Z^L^ in the cells.

Furthermore, it has been found that various potential sgRNAs exist in human plasma [15]. Among them, a 31/32-nt RNA fragment derived from 94-nt Y4-RNA is particularly noteworthy [16]. Although its genuine target RNAs are not known, the observations that it is overwhelmingly abundant in plasma and that it can function as sgRNA for tRNase Z^L^ have suggested that the 31/32-nt Y4-RNA fragment may work as an intercellular signaling molecule [17].

In addition, the observation that the white blood cell count and the C-reactive protein level show moderate positive correlations with the Y4-RNA fragment level in plasma has suggested that it could be used as a novel inflammatory marker [18]. It would be also notable that the Y4-RNA fragment levels of all four multiple myeloma patients examined were beyond the normal level even though their white blood cell counts were normal.

## 3. TRUE Gene Silencing

### 3.1. Four Types of sgRNA

In parallel with the studies on physiological aspects of tRNase Z^L^ and intracellular and extracellular sgRNAs mentioned above, the types of sgRNA and the interactions between tRNase Z^L^ and its RNA substrates have been intensively investigated and this has led to the development of the general RNA cleavage method [6,19,20,21,22,23,24,25]. With this method, any target RNA can be cleaved at any desired site by tRNase Z^L^ with the aid of appropriate sgRNA in a test tube (Figure 1). Incidentally, a similar method has been developed using RNase P and external guide RNA [25].

tRNase Z^L^ primarily recognizes the L-shaped folding structure formed from the cloverleaf-like secondary structure of pre-tRNA and cleaves it after the discriminator nucleotide to remove its 3′ trailer [4]. In the same fashion, a pre-tRNA-like RNA complex formed between a target RNA and a 5′-half-tRNA or 3′-truncated tRNA can be recognized by tRNase Z^L^ and the target RNA can be cleaved [13,19]. 

tRNase Z^L^ can also recognize and cleave micro-pre-tRNA that lacks the D, anticodon, and extra arms after a nucleotide corresponding to the discriminator as efficiently as pre-tRNA [24]. In similar ways, tRNase Z^L^ can recognize a micro-pre-tRNA-like RNA complex formed between a target RNA and a hook-shaped RNA, ~14-nt linear RNA, or heptamer RNA and cleave the target RNA [6,20,22,24].

On the basis of the above property of tRNase Z^L^, we can cleave a target RNA with various specificities in sequence and length by selecting appropriate guide RNAs. tRNase Z^L^ can work as an ~4-nt sequence specific RNA cutter with 3′-truncated tRNAs or hook-shaped RNAs, and as 12-nt and ~14-nt sequence specific RNA cutters with 5′-half-tRNAs and ~14-nt linear RNAs, respectively. Since an ~5-base-pair T-arm-like structure is needed for recognition of a target RNA by tRNase Z^L^ when using heptamer RNA, roughly speaking, the target RNA can be cleaved with an ~12-nt (not merely 7-nt) sequence specificity [20]. The last four guide RNAs are termed small guide RNA, or sgRNA for tRNase Z^L^ (Figure 1).

With respect to heptamer-type sgRNA, how chemical modifications on it affect its guiding efficiency has been investigated. The guiding efficiencies (*V_max_*/*K_m_*/*K_d_*) of the sgRNA with full 2′-*O*-methyl modifications, the sgRNA with full phosphorothioate modifications, sgDNA, and sgDNA with full phosphorothioate modifications have been shown to be 89, 53, 21, and 6%, respectively, of that of the natural sgRNA [26]. The efficiencies of heptamer-guided target RNA cleavage by tRNase Z^L^ have been shown to decrease as the number of LNA-modified riboses increases [27].

Furthermore, from the investigation on the interaction between tRNase Z^L^ and pre-tRNA, several pieces of information important for selecting sgRNA have been obtained. On the whole, the efficiency of pre-tRNA cleavage by tRNase Z^L^ decreases as its 3′ trailer becomes longer [28]. The cleavage efficiency for pre-tRNA with its 3′ trailer starting with G or A is the highest, while that for pre-tRNA with its 3′ trailer starting with C is the lowest [28]. The cleavage of pre-tRNA with its 5′ leader of more than 8 nt is severely inhibited, and that of pre-tRNA with its 5′ leader that forms more than 2 base-pairs with its 3′ trailer resulting in an extended acceptor stem is also suppressed [29].

Although tRNase Z^L^ usually cleaves pre-tRNA after the discriminator nucleotide, in some cases, it cleaves a pre-tRNA-like or micro-pre-tRNA-like complex at multiple sites near a nucleotide corresponding to the discriminator [19,20,22]. This would be due to the presence of multiple conformers of the RNA complex and/or the presence of multiple modes of the RNA complex/tRNase Z^L^ interaction. The experiments for pre-tRNA variants have shown that, in general, pre-tRNA variants containing a total of >11 base pairs in the acceptor stem and the T stem are cleaved only after the discriminator and that pre-tRNA variants with a total of N base pairs (N is <12) are cleaved 12 minus N and 13 minus N nt downstream of the discriminator [30].

As explained in detail above, tRNase Z^L^ can cleave target RNA with varying sequence/length specificities under the direction of appropriate sgRNA. Target RNA can be as small as miRNA and as long as mRNA, and any site can be targeted including a 3′-terminal nucleotide with the exception of tightly folded regions and 5′-terminal 4 sites. 

Target sites for each type of sgRNA can be chosen as follows. With respect to 5′-half-tRNA-type and heptamer-type sgRNA, first, hairpin structures resembling the T-arm of tRNA in a target RNA are selected with the aid of an appropriate computer program and/or visually. Then, candidate target sites are chosen from among them by excluding the sites in potentially tightly folded RNA regions, which can be predicted by a computer program for RNA secondary structure prediction. With respect to hook-type and 14-nt linear-type sgRNA, the first step is not needed. Although RNA primary structures of target sites seem to hardly affect RNA cleavage efficiency by tRNase Z^L^, some sequence constraints could be found by further analyses.

### 3.2. Modulation of Gene Expression by TRUE Gene Silencing

**Figure 2:**
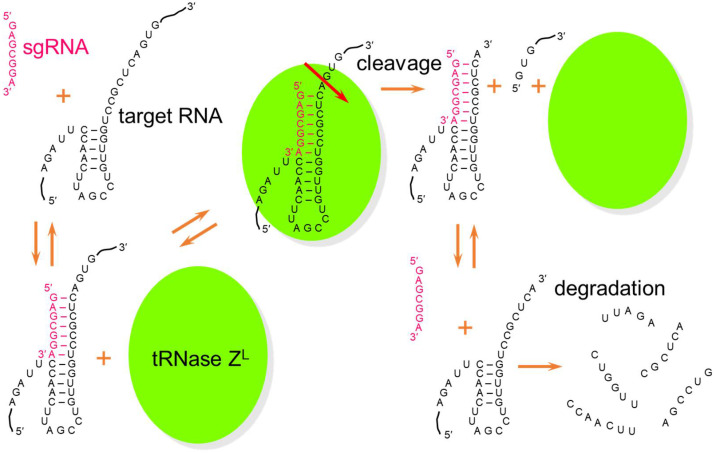
Molecular mechanism of TRUE gene silencing. A heptamer-type sgRNA binds a target RNA immediately after a 5-base-pair T-arm-like structure to form an sgRNA/target RNA complex. tRNase Z^L^ recognizes this RNA complex and cleaves target RNA after a nucleotide corresponding the discriminator. The cleaved RNAs are further degraded by other nucleases, and the sgRNA and tRNase Z^L^ can be reused to cleave another target RNA.

The efficacy of the general RNA cleavage method using tRNase Z^L^ and sgRNA in living cells has been examined by measuring expression levels of reporter genes and endogenous genes in cultured cells or in mice. As below, its efficacy has been shown with various degrees, and this technology is called TRUE gene silencing after tRNase Z^L^-utilizing efficacious gene silencing (Figure 2). Some of the examples are the application of TRUE gene silencing to several human cancer cells in order to induce apoptosis in them, and are listed in Table 1.

It has been demonstrated that the expression of chloramphenicol acetyltransferase can be downregulated by a 5′-half-tRNA-type sgRNA targeting its mRNA which is introduced into Madin–Darby canine kidney epithelial cells as an expression plasmid or a synthetic 2′-*O*-methyl RNA [26]. It has also been shown that 2′-*O*-methyl heptamer-type sgRNAs targeting luciferase mRNA variants can downregulate their mRNA levels in HEK293 cells and that a 2′-*O*-methyl heptamer-type sgRNA targeting the BCL2 mRNA reduces viability of Sarcoma 180 mouse cells [26].

It has been shown that HIV-1 gene expression is suppressed in COS cells by transiently expressing 5′-half-tRNA-type sgRNAs targeting the gag mRNA and that the expression of HIV-1 gag p24 is almost completely suppressed in Jurkat cells stably expressing a 5′-half-tRNA-type sgRNA targeting the gag mRNA for at least 18 days after challenge with HIV-1 [38].

The involvement of tRNase Z^L^ in downregulation of cellular mRNAs has been shown by the observation that the efficacy of luciferase downregulation by a 5′-half-tRNA-type or 14-nt linear-type 2′-*O*-methyl sgRNA targeting the luciferase mRNA increases with an increasing cellular tRNase Z^L^ level in HEK293 cells [39]. It has been observed that BCL2 and GSK3B expression can be downregulated in HEK293 cells that stably express a 5′-half-tRNA-type or 14-nt linear-type sgRNA targeting the BCL2 and GSK3B mRNAs, respectively [39]. Furthermore, the expression of chloramphenicol acetyltransferase and luciferase has been shown to be suppressed in the livers of postnatal mice that were tail-vein-injected with a 5′-half-tRNA-type 2′-*O*-methyl sgRNA targeting the chloramphenicol acetyltransferase mRNA and a heptamer-type 2′-*O*-methyl sgRNA targeting the luciferase mRNA, respectively [39].

To discover anti-angiogenic sgRNAs, TRUE gene silencing has been applied to the VEGF gene by using four 5′-half-tRNA-type sgRNAs and four 14-nt linear-type sgRNAs targeting the human VEGF mRNA [40]. It has been found that many of them downregulate the exogenous VEGF mRNA efficiently in HEK293 and HeLa cells and that two of them downregulate the endogenous VEGF gene expression in HeLa cells much more efficiently than an siRNA.

It has been shown that five naked heptamer-type sgRNAs with 2–7 LNA modifications targeting the BCL2 mRNA reduce its mRNA levels in Jurkat cells as efficiently as a heptamer-type sgRNA with no LNA modification [27].

It has been demonstrated that a naked 14-nt linear-type sgRNA targeting human miR-16 significantly reduces the miR-16 level in HEK293 and HL60 cells [41]. Three other naked 14-nt linear-type sgRNAs targeting miR-142-3p, miR-206, and miR-19a/b have also been shown to downregulate the respective miRNA levels in various mammalian cell lines [41]. It appears that, in general, a specific cellular miRNA can be eliminated at least by ~50% by using a naked linear-type sgRNA.

A naked heptamer-type sgRNA targeting the human BCL2 mRNA has been shown to moderately downregulate its mRNA level in the human HL60 leukemia cells and to induce apoptosis in them (Table 1) [32]. In xenograft experiments using nude mice implanted with the HL60 cells, it has been shown that the median survival of the mice treated with this sgRNA is longer than that of the control mice [32].

It has been demonstrated that naked heptamer-type sgRNAs targeting the WT1 mRNA can reduce its mRNA levels and WT1 protein amounts in the WT1-expressing leukemia cells and that these sgRNAs efficiently induce apoptosis in these cells but not in WT1-nonexpressing cells (Table 1) [33]. 

To search for potential therapeutic sgRNAs for leukemia, a library composed of 156 heptamer-type sgRNAs has been screened, and 39 sgRNAs have been found to be able to efficiently induce apoptosis in human HL60 leukemia cells (Table 1) [34,35,42]. Furthermore, it has been demonstrated that 4 of the 39 sgRNAs can reduce growth rates of HL60 cells in mouse xenograft model experiments [34].

To find potential sgRNA therapeutics for head and neck squamous cell carcinoma, six heptamer-type, two linear-type, and two 5′-half-tRNA-type sgRNAs targeting the human CCND1 mRNA have been examined (Table 1) [31]. It has been demonstrated that all of these sgRNAs downregulate the mRNA level in HSC-2 or HSC-3 cells and that a subset of the sgRNAs reduce its protein level and induce apoptosis in HSC-3 cells [31]. 

In the course of an experiment to investigate the ability of a heptamer-type sgRNA targeting the OCT4 mRNA to differentiate human amnion stem cells, it has been observed unexpectedly that the amnion cells exhibit a morphology resembling initialized cells [43]. Further examination of its effect on human HL60 leukemia cells has shown that this OCT4-mRNA-targeting sgRNA can upregulate the OCT4 expression and the expression of alkaline phosphatase in the cells [43]. Although nothing is known about the molecular mechanism of the OCT4 upregulation, the sgRNA might happen to suppress genes that suppress the OCT4 expression.

### 3.3. Potential sgRNA Therapeutics for Multiple Myeloma

To explore the possibility of sgRNA as therapeutic agents, multiple myeloma was chosen as a target disease, and an intensive investigation was conducted. Multiple myeloma is a refractory plasma cell cancer [44]. In spite of various treatment modalities [45,46], achieving complete remission appears to be very hard because drug-resistant mutations emerge almost inevitably in the course of myeloma cell evolution resulting in relapse of myeloma in most patients.

To search for potential sgRNA therapeutics for multiple myeloma, the library of 156 heptamer-type sgRNAs has been screened, and 7 and 5 sgRNAs have been shown to be able to efficiently induce apoptosis in human KMM-1 and RPMI-8226 myeloma cells, respectively (Table 1) [34,35,42].

It has been demonstrated that two consecutively aligned heptamer-type sgRNAs targeting the human BCL2 mRNA can guide target RNA cleavage by tRNase Z^L^ as efficiently as a corresponding 14-nt linear-type sgRNA and that the double heptamer-type sgRNA, but not the 14-nt linear-type sgRNA, downregulates the BCL2 mRNA in HL60 cells [27]. It has been shown that the double heptamer-type sgRNA and each single heptamer-type sgRNA reduce viability of RPMI-8226 and KMM-1 cells and also suppress the growth of KMM-1 cells in a mouse xenograft experiment (Table 1) [36]. The absence of stable downregulation of the BCL2 mRNA has suggested that the double heptamer-type sgRNA targets other cellular RNAs to reduce the myeloma cell viability. Furthermore, two sets of double heptamer-type sgRNA targeting the human CCND1 mRNA have been shown to synergistically reduce RPMI-8226 cell viability [36].

The interplay between the bone marrow microenvironment and myeloma cells is essentially important for the pathology. To search for heptamer-type sgRNAs that can shift tumor-associated macrophages toward the M1 state, a heptamer-type sgRNA library has been screened for the ability to upregulate IL-12b gene expression in human macrophage-like cell lines, and three such sgRNAs have been found [37]. It has been demonstrated that one of the sgRNAs also shows such ability in fresh human macrophages and mouse macrophage-like cell lines and efficiently suppresses human KMM-1 myeloma cell growth in immunocompromised mice that is deficient in acquired immunity (Table 1) [37]. The observation that this sgRNA cannot induce apoptosis in KMM-1 myeloma cells suggests that it affects mouse macrophages in the tumor microenvironment and induces them to eliminate the implanted myeloma cells.

With respect to the above two sgRNAs that worked efficiently in mouse experiments [36,37], in the next stage, cellular target RNAs of these two sgRNAs need to be identified by transcriptome analyses to elucidate molecular mechanisms for how these sgRNAs work. In parallel, the efficacy of these two sgRNAs in mouse xenograft experiments needs to be examined by their systemic administration rather than local administration. 

Although the development of sgRNA therapeutics for multiple myeloma is still in its infancy, there are several advantages worth mentioning: sgRNA can be taken up by cells without any transfection reagent [32,33]; since tRNase Z^L^ exists ubiquitously in a human cell, in theory, any cellular RNA in cytosol, nucleus, and mitochondria can be targeted [7]; since discontinuous capillaries are formed in bone marrow, myeloma is one of the best targets of sgRNA therapeutics from the viewpoint of drug delivery. Concerning the first advantage, the cellular uptake of naked sgRNA is thought to occur by endocytosis, although the molecular mechanism is not fully understood [47]. 

Since the above two sgRNAs were obtained from screenings of libraries composed of only less than 160 heptamer-type sgRNAs, much better heptamer-type sgRNAs would be expected to be discovered from a screening of the full library composed of 16,384 heptamer-type sgRNAs. In parallel, by examining 5′-half-tRNA-type and 14-nt linear-type sgRNAs designed to target appropriate mRNAs such as the BCL2, CCND1, and IRF4 mRNAs for their ability to induce apoptosis in myeloma cells, potential sgRNA therapeutics would be expected to be found. Further nucleotide modifications of sgRNAs in addition to the 2′-*O*-methyl modification would also improve their efficacy in inducing apoptosis in myeloma cells. Lastly, since heptamer-type, 5′-half-tRNA-type, and 14-nt linear-type sgRNAs consist of 16,384, 16,777,216, and 268,435,456 different sequence sgRNAs, respectively, sgRNA therapeutics may help promote personalized medicine by selecting the most appropriate sgRNA depending on a patient’s own mRNA sequence and changing patient conditions. 

## Figures and Tables

**Table 1:** Application of TRUE gene silencing to human cancer cells in order to induce apoptosis.^1.^

Cancer	Target Cell	Target mRNA ^2^	sgRNA Type	Experimental Type ^3^	Reference
head and neck squamous cell carcinoma	HSC-2 HSC-3	CCND1	Heptamer 14-nt linear 5′-half-tRNA	in vitro	[31]
leukemia	HL60 C2F8	BCL2 WT1 screening	heptamer	in vitro in vivo	[32,33,34,35]
multiple myeloma	KMM-1 RPMI-8226	BCL2 CCND1 screening	heptamer	in vitro in vivo	[34,35,36]
	THP-1 U937 fresh monocyte J774.1 (mouse) RAW264.7 (mouse)	screening	heptamer	in vitro in vivo	[37]

^1^ The last example is the application to macrophage-like cells in order to shift them toward the M1 state. ^2^ “screening” denotes that target RNAs of the sgRNAs, which were obtained by screening of sgRNA libraries, are unknown. ^3^ “in vitro” and “in vivo” denote cultured cell experiments and mouse xenograft experiments, respectively.

## Data Availability

Not applicable.
